# Hybrid Brain/Neural Exoskeleton Enables Bimanual ADL Training in Routine Stroke Rehabilitation

**DOI:** 10.1161/STROKEAHA.125.052008

**Published:** 2025-11-17

**Authors:** Annalisa Colucci, Mareike Vermehren, Cornelius Angerhöfer, Niels Peekhaus, Won-Seok Kim, Won Kee Chang, Volker Hömberg, Nam-Jong Paik, Surjo R. Soekadar

**Affiliations:** Clinical Neurotechnology Laboratory, Department of Psychiatry and Neurosciences, Charité Campus Mitte (CCM), Charité–Universitätsmedizin Berlin, Germany (A.C., M.V., C.A., N.P., S.R.S.).; Department of Rehabilitation Medicine, Seoul National University College of Medicine, Seoul National University Bundang Hospital, Seongnam-si, Gyeonggi-do, Republic of Korea (W.-S.K., W.K.C., N.-J.P.).; Department of Neurorehabilitation, SRH Gesundheitszentrum Bad Wimpfen GmbH, Germany (V.H.).

**Keywords:** activities of daily living, brain-computer interface, electrooculography, electroencephalography, motor disorders, stroke

## Abstract

**BACKGROUND:**

Severe upper limb motor impairment is one of the most disabling consequences of stroke. Although brain-controlled rehabilitation technologies, such as brain/neural exoskeletons (B/NE), have been shown to be effective in promoting motor recovery, their clinical adoption remains limited because of insufficient integration of B/NE into existing clinical workflows. Here, we introduce and validate a fully portable B/NE system that overcomes this limitation by relying on brain (electroencephalography) and ocular (electrooculography) signals to restore bimanual activities of daily living within a novel therapeutic framework.

**METHODS:**

In this pilot study, we tested the feasibility of the novel approach in 5 stroke survivors (mean age, 51 years; SD=14.8) undergoing inpatient neurorehabilitation. Stroke survivors aged 18 to 80 years, who exhibited hemiparesis and sufficient cognitive ability to understand and follow instructions, were invited to participate in a 1-hour training session. This session included system setup and calibration, followed by performing B/NE–supported, self-paced bimanual activities of daily living. As primary outcome measures, we assessed control accuracy, the ability to reliably modulate electroencephalography and electrooculography signals, and time to initialize, defined as the time required to react to cues and initiate the task, serving as a measure of control intuitiveness. In addition, participants’ B/NE control performance during assisted training of bimanual activities of daily living, as well as setup preparation time, were assessed via direct observation.

**RESULTS:**

Participants demonstrated reliable control accuracy in using both brain (mean, 83%; SD=15.36) and ocular (mean=100%) signals, as well as intuitive control (time to initialize <2 s). All participants reliably controlled the B/NE performing a battery of 10 bimanual activities of daily living. Moreover, setup and calibration times remained below 20 minutes.

**CONCLUSIONS:**

These findings highlight the compatibility of the novel B/NE with existing clinical workflows and its feasibility to enable B/NE–supported stroke neurorehabilitation by facilitating seamless integration into clinical practice.

Stroke is the main cause of adult long-term disability.^1^ Currently, broadly accessible treatment options that restore motor functions in stroke survivors with severe motor impairment, that is, without voluntary extension of wrist and fingers, are lacking.^2^ Over the past decade, brain-computer interfaces (BCIs) translating neural activity into control commands of external devices have been developed, triggering neuroplasticity^3^ and motor recovery,^4^ in both acute and chronic stroke survivors.^5^ Such rehabilitative motor BCIs can fill the gap in available treatment options in severe paralysis^2,6^ by reestablishing a direct communication between the brain and the paralyzed limb with the support of a robotic orthosis. By restoring hand and arm movements, for example, brain/neural exoskeletons (B/NE) could enable severely affected stroke survivors to perform bimanual activities of daily living (ADLs) during physiotherapy.^7^

Despite robust evidence supporting BCI efficacy with a moderate effect size,^5^ their integration into routine clinical care remains limited^8^ due to technical challenges, including control accuracy (CA) and intuitiveness,^9^ as well as issues related to compatibility with clinical workflows, such as minimal setup time^10^ and patient engagement.^11,12^ CA, that is, reliability in translating user intention into commands, is highly dependent on signal quality and compromised by signal artifacts, particularly in out-of-the-lab environments. However, high contingency between neural activity and proprioceptive feedback is crucial for operant learning.^13,14^ Intuitiveness, that is, ease of use without complicated instructions, is critical as many stroke survivors suffer from early fatigue^15^ and cognitive impairments.^16^ Minimal setup and calibration times are also important, given the 45- to 60-minute therapy window. Moreover, applying rules for learning-oriented motor therapy,^17^ for example, high repetition, active behavior, ecological validity, and motivation, is essential to maximize motor recovery.

To address these factors, we developed a novel therapeutic framework that builds on a fully portable hybrid B/NE using brain and ocular signals and tested it in 5 stroke survivors. CA, intuitiveness, setup time, and ability of patients to engage and complete self-paced, bimanual ADLs were assessed, and compatibility with clinical workflows was validated.

## Methods

### Participants

The study was approved by the local ethics committee at Charité–Universitätsmedizin Berlin under the number EA1/106/20. CONSORT reporting guidelines (Consolidated Standards of Reporting Trials) for feasibility trials were applied.^18^ We recruited participants at SRH Gesundheitszentrum Bad Wimpfen and P.A.N. Zentrum Berlin-Frohnau, Germany. Stroke survivors aged 18 to 80 years undergoing inpatient neurorehabilitation for hemiparesis and with sufficient cognitive ability to follow instructions were screened. To maximize generalizability, we opened the study to hemorrhagic and ischemic stroke survivors, both in subacute and chronic phases. A detailed description of the inclusion criteria is provided in the Supplemental Material. Eligible participants were B/NE naive, continued standard rehabilitation, and provided written informed consent for participation and video recording. Data and videos of participants’ performance are available on request.

### B/NE System

**Figure 1.**
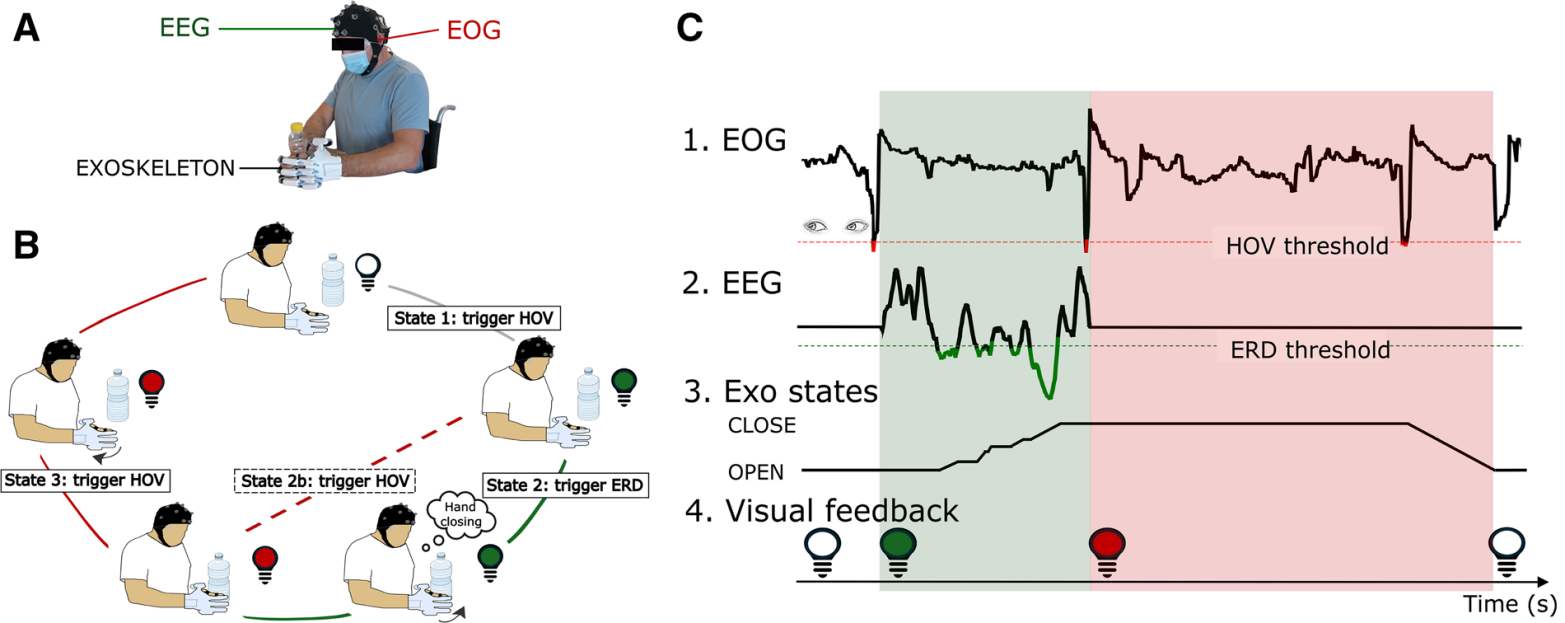
**Design of the hybrid brain/neural exoskeleton (B/NE). A**, Representation of the B/NE setup, including a portable semidry electroencephalography (EEG) amplifier acquiring both brain and ocular (electrooculography [EOG]) signals, and a portable robotic hand exoskeleton. **B**, Scheme of the novel sequential B/NE control strategy. At the start, a white light indicated the B/NE system's readiness for use. A maximal lateral eye movement, that is, horizontal oculoversion (HOV), activated brain control (on mode). At this state, the system became sensitive to both event-related desynchronization (ERD) of sensorimotor rhythms (SMR) to close the exoskeleton as well as HOV to stop the motion, in case of sufficient object hold or unwanted exoskeleton motions. Once the hand was fully closed or blocked, the light turned red, indicating that brain control was turned off. At this state, participants could freely manipulate objects to perform bimanual tasks. Finally, a new HOV opened the exoskeleton. Once the robotic hand was fully open, the light turned white again, starting a new control cycle. **C**, Exemplary trial to show timing and contingencies between real-time classification of HOV (1), SMR-ERD (2), exoskeleton states (3), and visual feedback (4) during the sequential control paradigm. When the EOG threshold (red dotted line) was crossed, an HOV was detected, and the state of the control changed. In the green state, when the ERD threshold (green dotted line) was crossed, the intention to move was detected and translated into exoskeleton closing motions.

A portable B/NE system was used for real-time detection and classification of brain and ocular signals to control a robotic hand exoskeleton (Figure 1A). All biosignals were recorded using a wireless saline-based electroencephalography system and processed in near real-time (<100 ms) via a custom BCI software (BeamBCI). A detailed description of biosignal recording and processing is presented in the Supplemental Material.

Electroencephalography activity was recorded via a minimal setup using 5 recording sites.^19^ Downmodulation of sensorimotor rhythms (SMR) occurring during movement imagination/attempt, as measured by event-related desynchronization (ERD)^20^ was evaluated in real-time.

Electrooculography was recorded via 2 additional electrodes placed over the outer canthi. Maximal horizontal oculoversions (HOVs)^21^ induce a strong modulation in the electrooculography signal, which can serve as a reliable signal for real-time control.^22,23^

SMR-ERD and HOV were integrated into a sequential control paradigm (Figure 1B) that maximizes accuracy in a realistic environment and allows self-paced B/NE–supported motor training. SMR-ERDs, occurring during imagination/attempted hand closing motions, were translated into exoskeleton closing motions. Visual feedback was used to indicate the different control states (Figure 1B). HOVs directed to the exoskeleton’s contralateral side were either translated into exoskeleton activation (on mode, depicted in Figure 1C as green interval) or stop commands in case of unintentional exoskeleton closing motions (veto). After successful object manipulation (eg, at the end of the red interval depicted in Figure 1C), HOV were used to initiate exoskeleton opening motions. A more detailed description is presented in the Supplemental Material.

For the evaluation of the B/NE paradigm, participants were equipped with a wearable hand exoskeleton (HandyRehab, Zunosaki Ltd, Hong Kong), donned on the paretic hand. A minimum of 4 s SMR-ERD required to perform a stable grasp, ensured patient’s engagement and sustained contingent feedback.

### Experimental Setup: Calibration and Testing of the B/NE System

**Figure 2.**
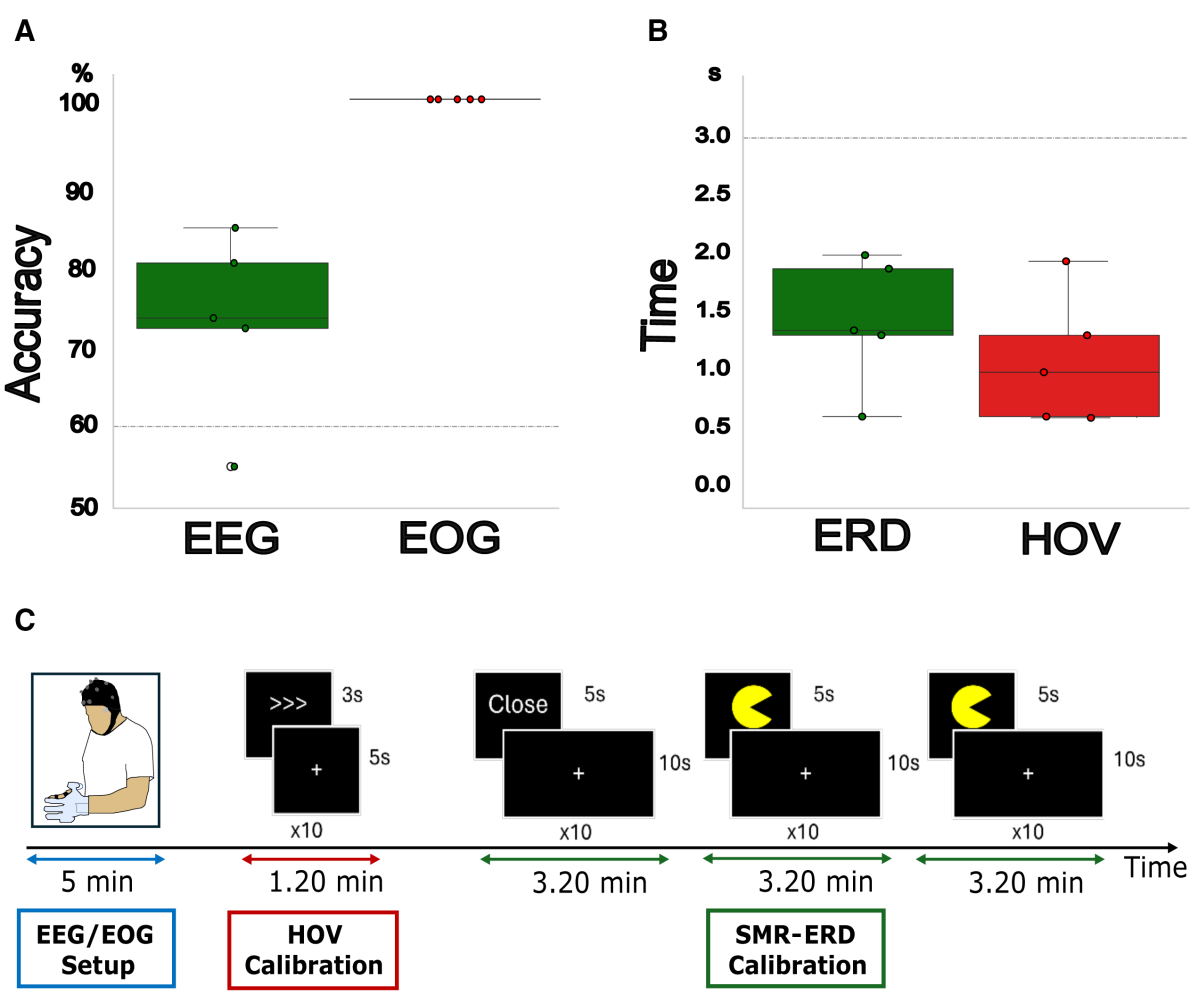
**Evaluation of the brain/neural exoskeleton (B/NE) control and calibration. A**, Control accuracy, evaluated as percentage of correctly classified trials in the electroencephalography (EEG) and electrooculography (EOG) signals. Participants achieved an average control accuracy of 83% (SD=15.36) over the EEG and a perfect accuracy over the EOG (100%), proving the ability to establish a reliable and consistent control over the 2 biosignals (>60%). **B**, Control intuitiveness, estimated as the time to initialize an event-related desynchronization (ERD) of sensorimotor rhythms (SMRs), by imagining/attempting hand movements, or to perform a maximal horizontal oculoversions (HOV). On average, participants initiated ERDs in 1.65 s (SD=0.68) and HOVs in 0.74 s (SD=0.11), confirming the high intuitiveness of both tasks (<3 s). **C**, Schematic representation of the B/NE’s setup and calibration paradigm and its duration. We envisioned and tested the feasibility of a novel pipeline to minimize the setup and calibration time of our B/NE system in a clinical environment. First, participants are equipped with the portable and wireless saline-based EEG system, requiring about 5 minutes. Then, the EOG signal is assessed to calibrate the individual HOVs detection threshold. Finally, the assessment of the EEG signal and the calibration of the threshold to classify ERD of SMRs is performed. The total operationalization of the novel B/NE system can be achieved in <20 minutes.

To evaluate the feasibility of the novel B/NE, participants were invited for a 1-hour training session. Participants were seated comfortably in front of a table and were equipped with the wireless biosignal recording device. To start calibration, we assessed the electrooculography signal and computed the individual HOV detection threshold. Then, the SMR-ERD calibration was performed, during which participants were trained to attempt small grasping motions or quasi movements^24^ of the paretic hand, or to relax. A schematic representation of the calibration is illustrated in Figure 2C, and a more detailed description is presented in the Supplemental Material. At the end of the calibration, participants were equipped with the exoskeleton, familiarized with the sequential control, and finally instructed to perform a battery of 10 different bimanual ADLs, to simulate therapy with the support of the exoskeleton. The description of all bimanual ADLs is presented in the Table.

**Table.**
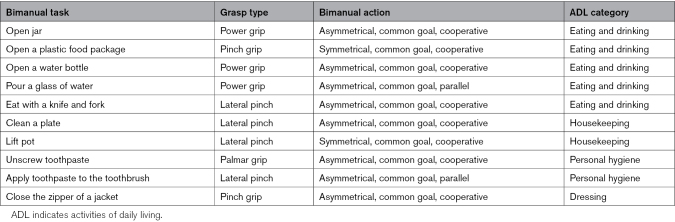
Description of the Battery of 10 Bimanual ADLs, Categorized by Grasp Type, Kantak Grasp Taxonomy, and ADL Category

### Outcome Measures

CA was evaluated based on electroencephalography and electrooculography signals acquired during the last run of SMR-ERD and HOVs calibration, containing at least 5 trials per condition per participant. Thus, CA evaluates the success of the calibration and infers the ability of participants to control the B/NE system. As established in the BCI field, CA was defined as the ratio between correctly classified trials and the total number of trials.^9^ An electroencephalography trial was considered correctly performed if the participant successfully downmodulated neural activity during movement trials—defined as a reduction in SMR activity below the 75th percentile—while maintaining higher SMR levels during rest periods. CA was evaluated based on the last 4 s of each 5-s–long activation interval, and the central 4 s of an intertrial interval at which participants were at rest. A successful electrooculography trial was defined as a modulation above the 75th percentile of the ocular signal. In analogy to SMR-ERD, the electrooculography CA was computed as the ratio between correctly classified and the total number of trials.

Time to Initialize was assessed, that is, participants’ time to react to cues and voluntarily modulate brain and ocular signals during calibration, as an estimate of biosignal control intuitiveness. Intuitive control was defined as the initiation of biosignal modulation, that is, reaching 50% of the maximal ERD or HOV, within <3 s after cue presentation.^21,25^

Quantifying the accuracy and performance of asynchronous, self-paced B/NE control during ADL training is challenging due to the absence of predefined experimental cues. Therefore, we evaluated the successful performance of bimanual ADLs via direct observation of the B/NE training. As a representative example, a video of a control cycle is provided (Video S1), demonstrating precise timing and contingencies between real-time classification of HOV, SMR-ERD, visual feedback, and exoskeleton motions (Figure 1C). Moreover, the B/NE setup was video-taped in 1 representative participant (Video S2), demonstrating the average preparation time for biosignal recordings.

## Results

Out of 7 individuals screened, 5 B/NE–naive stroke survivors were recruited (mean age, 51 years; SD=14.8). The sample included 3 hemorrhagic and 2 ischemic stroke survivors, 3 of them being in the subacute phase and 2 in the chronic phase. A more detailed description is provided in Table S1. None of the participants reported harms or unintended consequences.

### Accuracy and Intuitiveness of the Novel B/NE System

The evaluation of B/NE calibration showed that, on average, participants reached a CA of 83% (SD=15.36) during electroencephalography control (Figure 2A). All participants reached a CA of 100% (SD=0) during electrooculography control (Figure 2B).

Evaluation of time to initialize showed that participants were able to downmodulate SMR within 1.65 s (SD=0.68) on average after cue presentation (Figure 2), and perform HOVs within 0.74 s (SD=0.11), demonstrating intuitive control during both tasks.

Finally, all participants understood the sequential control and could reliably control the hybrid B/NE system to perform, within the first session, a battery of 10 bimanual ADLs, confirming the feasibility of the B/NE–supported training.

### B/NE Setup and Calibration

Time for mounting and calibrating the B/NE system ranged on average below 20 minutes and included one run of HOV calibration lasting ≈1.20 minutes and 3 runs of SMR-ERD calibration lasting in total about 10 minutes (Figure 2C). A more detailed description of the B/NE calibration can be found in the Supplemental Material.

## Discussion

Despite robust evidence supporting the efficacy of BCI-based treatments for severe stroke, clinical adoption remains limited due to insufficient integration of B/NEs into existing clinical workflows.

This is a critical shortcoming in the field of rehabilitation, as alternative treatments for complete finger paralysis are lacking. To address this, our study introduced a fully portable B/NE system designed to restore bimanual ADLs, thereby expanding available rehabilitation options in stroke with severe finger paralysis.

Our findings confirm the system’s reliability, with CA above 70%, facilitating Hebbian plasticity for motor recovery. Participants could modulate brain and ocular signals within 2 s, demonstrating ease of use and rapid engagement.

Notably, all B/NE–naive participants successfully operated the system and performed ADLs in their first session, highlighting its accessibility and clinical integration potential.

A key advantage of our system is the self-paced initiation of movement, enhancing engagement and ecological validity. By focusing on bimanual ADLs, the system boosts motivation and promotes high-repetition training, supporting real-life skill transfer. In addition, setup and training can be completed within 60 minutes, demonstrating the system’s usability within existing clinical workflows. Compared with previous work on B/NEs,^26–28^ we show that integrating brain-controlled technology into clinical practice is not only feasible but also compatible with routine care. Importantly, this study introduces the first protocol for incorporating B/NE–supported physiotherapy and occupational therapy into standard rehabilitation programs.

Although all participants in this pilot study were able to operate the BCI and perform ADLs, these results should be considered alongside specific limitations, primarily the small sample, including relatively young patients with none or only a few comorbidities. Future work needs to evaluate the generalizability of the novel approach, enlarging the sample to cover the full spectrum of stroke types, locations, and clinical outcomes, as well as its clinical efficacy, with larger randomized clinical trials. Moreover, future iterations should address more stratified intervention strategies that account for common stroke complications, for example, adaptable control mechanisms for unreliable electrooculography signals, and incorporate multitask training paradigms.

In conclusion, our study demonstrates the feasibility of a novel B/NE approach to stroke rehabilitation that demonstrates high reliability, intuitive control, and seamless integration into clinical workflows. By enabling rapid setup and operation with minimal training, our system addresses key usability barriers while laying the foundation for future B/NE systems that comply with US Food and Drug Administration and Medical Device Regulation certification requirements, such as safety and performance validation, usability testing, and clinical evaluation. This positions the technology for large-scale clinical trials and accelerates its path toward widespread clinical adoption. To fully realize its potential, future efforts should focus on enhancing accessibility, streamlining regulatory pathways, securing long-term clinical validation, and examining neuroethical dimensions.^29^

## ARTICLE INFORMATION

### Acknowledgments

The authors extend their gratitude to the staff of SRH Gesundheitszentrum Bad Wimpfen and P.A.N. Zentrum Berlin-Frohnau and the study participants for their valuable contributions to this study. A. Colucci had full access to the data and takes responsibility for its integrity and data analysis.

### Sources of Funding

This research was supported in part by the European Research Council (ERC) under the project Next-Generation Brain-Machine Interfaces (NGBMI; 759370) and BNCI2 (101088715), by the Federal Ministry of Research and Education (BMBF) under the projects SSMART (01DR21025A) and NEUROSTAR (01DR25007A), by the Einstein Foundation Berlin, and by the German Research Foundation (DFG) under the project NEURO-EXO (SO932/7-1).

### Disclosures

None.

### Supplemental Material

Supplemental Methods

Table S1

Videos S1–S2

Major Resources Table

CONSORT Checklist

## Supplementary Material


