# Supplementary material for: Hybrid Brain/Neural Exoskeleton Enables Bimanual ADL Training in Routine Stroke Rehabilitation
Source: Stroke. 2025 Nov 17;57(2):505–10. doi: 10.1161/STROKEAHA.125.052008 (PMC12829497; doi:10.1161/STROKEAHA.125.052008)
Supplement: Supplementary file 1 [file str-57-505-s001.pdf]

Supplemental material

Supplemental methods

Participants recruitment

We recruited participants at SRH Gesundheitszentrum Bad Wimpfen and P.A.N. Zentrum Berlin-Frohnau, Germany. All stroke survivors undergoing inpatient neurorehabilitation at the time of the visits were screened for the study. Seven participants were screened and five were finally recruited according to the following inclusion criteria: suffered from ischemic or hemorrhagic stroke, subacute or chronic stage of recovery with upper-limb hemiparesis, age ranging between 18 and 85 years, cognitive ability to understand and follow instructions and absence of neurological or psychiatric conditions other than stroke affecting their ability to understand and/or control the brain/neural exoskeleton (B/NE) system. Two participants were excluded due to secondary complaints after stroke, namely cognitive impairment and diminished control over eye movements.
All participants were B/NE naïve and were yet undergoing intensive rehabilitation. Criteria of stroke characterization were left rather broad to investigate the clinical applicability of the novel hybrid B/NE across a diverse sample of stroke survivors and ensure the maximal generalizability of our results, despite the limited sample size. The sample of participants was indeed quite heterogeneous, including both hemorrhagic and ischemic stroke, as well as subacute and chronic survivors, as can be seen in table S1. Also, severity of the upper limb paresis was quite diverse, as indicated by the ability to use the affected hand, as assessed by the subscale 7 of the Stroke Impact Scale and the ability to perform bimanual tasks, as assessed by the CAHAI-8. Considering the feasibility nature of the study and the high resource demands associated with clinical site visits, the small but diverse sample was considered sufficient.

Biosignals preprocessing

All biosignals were amplified with a portable and wireless saline-based electroencephalography (EEG) system (smarting®, mBrainTrain®, Belgrade, Serbia, in combination with a customized GT Gelfree-S3 EEG cap, Wuhan Greentek Pty Ltd, Wuhan, China) and sampled at 250 Hz. Using saline-soaked sponges, the system reduces preparation time and eliminates the need for hair washing after recording, as opposed to gel-based EEG, a great burden for users affected by paresis. A custom brain computer interafce (BCI) software (BeamBCI) was employed for real-time signal processing and classification. EEG activity was recorded via a minimal setup including 5 conventional recording sites, either over the right (F4, T8, C4, P4 and Cz) or the left motor cortex (F3, T7, C3, P3 and Cz), contralateral to the paretic arm. A reference electrode was located over FCz and a ground electrode over Fpz. EEG signal was bandpass-filtered between 1 and 30Hz and the signal over C3 and C4 was Laplace-filtered to optimize signal-to-noise ratio of sensorimotor rhythms (SMR), i.e., motor-

related brain oscillations in the range between 8-13 Hz, while minimizing competing activity from distant sources. Instantaneous power of SMR was then estimated based on the Burg algorithm for a 3 Hz wide bin around the individual peak SMR frequency[19] within sample blocks of 400ms. Downmodulation of alpha SMR during movement imagination/attempt, i.e., event related desynchronization (ERD), was estimated according to the method introduce by Pfurtscheller and Aranibar[18]. ERD were translated in the closing motion of the visual feedback during calibration or of the exoskeleton during the activities of daily living (ADLs) training, when a downmodulation below the average EEG signal during trials of movement attempt occurred[19]. Thus, EEG classification and feedback presentation were updated at a minimum rate of 10Hz, providing near real-time, continuous assessment of brain activity to participants. Electrooculography (EOG) was recorded via 2 additional electrodes over the left and right outer canthi. We estimated bipolar EOG signal by subtracting left from right EOG and later bandpass filtered the signal between 0.02-3 Hz. Maximal horizontal oculoversions (HOVs), inducing a strong curve shape modulation in the EOG signal, is a pronounced and reliable signal for real time interfacing. Such maximal oculoversions, performed while holding the head straight towards the screen in front of the user, is a movement rarely occurring in everyday life. This maximizes the specificity of the HOV signal to signify intentional oculoversions, while minimizing the likelihood of false detection of more frequent eye movements. Thus, HOVs contralateral to the hand exoskeleton, with a modulation of over 70% of the median maxima EOG signal[19], were translated into exoskeleton activation, stop command for unintentional closing or opening movements.

B/NE system control strategy

The neural and ocular bio signals were integrated in a hybrid, sequential control strategy (figure 1B), to enable participants to engage in a self-paced B/NE-supported motor training. Indeed, HOVs, more reliable to detect and more robust to motion artifacts, were used to initialize the start of a new trial, activating the B/NE and its sensitivity to SMR-ERD classification in the EEG signal. This way, participants were not cued to perform a motor task, but self-paced the training, ensuring users' engagement and maximizing EEG accurate classification of voluntary SMR modulation. HOVs were used also to move throughout the sequential control's stages. Indeed, at start, a white light indicated the readiness of the B/NE system and the sensitivity to EOG-HOV signal, that could now switch the system to the green mode and "turn on" the brain control. At this stage, the system was sensitive to both SMR-ERD to close the exoskeleton and to HOV to stop the motion, in case of sufficient grasping or unwanted motion. Once the hand was fully closed or it had been blocked, the red light communicated to participants that the exoskeleton could not close any further and they could manipulate the object to perform bimanual ADLs. Finally, a new HOV could lead to a full opening of the exoskeleton. Once the exoskeleton was fully open, the light went back to white, and the user could start a new trial.

B/NE system calibration and bimanual ADLs training

At the beginning of each training session, a short calibration is needed to optimize the B/NE system to every participant's neural and ocular activity and optimize control accuracy. However, in clinics, time for set-up and calibration need to be minimized, in order to maximize the time dedicated to the bimanual training. First, participants were equipped with the portable and wireless saline-based EEG system. Relying on a minimal set-up (5 EEG electrodes, 2 EOG electrodes, ground and reference), the system was ready to use in less than 5 minutes (see video S2). Then, to start the calibration, we assess the EOG signal and compute the individual HOV detection threshold. Participants are instructed to perform HOVs to the side contralateral to the paretic hand and based on the recorded data, the threshold is computed. Afterwards, three runs of SMR-ERD calibration are performed, each alternating motor tasks (5 seconds) and inter-trial intervals (ITI) for relaxation (15 seconds). The aim of the first run is to have an initial estimate of peak SMR and ERD threshold, thus no feedback is provided. During the second and the third run instead, visual feedback in the form of a moving Pacman is introduced, which, based on the previously calibrated threshold, closes its mouth, as reinforcement of correct SMR-ERD detection. A schematic representation of the calibration is illustrated in figure 2C. At the end of each calibration run, peak frequency and detection threshold are updated. At the end of the calibration, participants were equipped with the exoskeleton, familiarized with the sequential control and finally instructed to perform a battery of 10 different bimanual ADLs. We introduced different objects and different type of manipulations to simulate what therapy with the support of the exoskeleton could look like. In Table 1 in the main manuscript, the description of all bimanual ADLs, including the grasp type required, the bimanual action according to the taxonomy of Kantak et al.[24] and the ADL category is shown.

Supplementary table

Patient ID	Age	Gender	Time Since Stroke	Stroke Location	Stroke Etiology	Stroke Impact Scale (sub scale 7)	CAHAI-8
ID_01	42	M	1 Month	Subcortical	Hemorrhagic	11/25	47/58
ID_02	40	M	4 Years	Subcortical	Ischemic	13/25	19,4/58
ID_03	76	M	3 Months	Subcortical	Hemorrhagic	7/25	14/58
ID_04	60	M	3 Months	Subcortical	Ischemic	5/25	47/58
ID_05	37	M	1,5 Year	-	Hemorrhagic	5/25	17/58

Table S1 Participants characteristics. *The table provides an overview of the participants' characteristics and their motor impairment. We report severity of the upper limb paresis according to the subscale 7 of the Stroke Impact Scale, assessing the ability to use the affected hand, and the ability to perform bimanual tasks, as assessed by the CAHAI-8.*

Legend for videos

Video S1 Brain/neural exoskeleton (B/NE) control strategy demonstration. A demonstrative trial showcasing the precise timing and contingencies between the real-time classification of horizontal oculoversions (HOV) in the electrooculography (EOG) signal, event related desynchronization (ERD) in the electroencephalography (EEG) signal, exoskeleton states, and visual feedback during sequential control. The participant utilizes the novel B/NE control to open a water bottle and pour water into a glass.

Video S2 Setup preparation. Preparation of the saline-based EEG system on a representative participant, with the full setup completed in approximately three minutes.

Major Resources Table

Data & Code Availability

Description	Source / Repository	Persistent ID / URL
Git Repository with code for offline data analyses	GIT	https://github.com/AnnalisaCol/BNE-evaluation.git
